# Influence of Solid Lubricant Addition on Friction and Wear Response of 3D Printed Polymer Composites

**DOI:** 10.3390/polym13172905

**Published:** 2021-08-28

**Authors:** R. Keshavamurthy, Vijay Tambrallimath, Ali A. Rajhi, Shabbir Ahmed R. M, Arun Y. Patil, T. M. Yunus Khan, R. Makannavar

**Affiliations:** 1 Department of Mechanical Engineering, Dayananda Sagar College of Engineering, Bengaluru 560078, India; rahulmakannavar088@gmail.com; 2 Department of Automobile Engineering, Dayananda Sagar College of Engineering, Bengaluru 560078, India; vijay.tambrallimath@gmail.com; 3 Department of Mechanical Engineering, College of Engineering, King Khalid University, Abha 61421, Saudi Arabia; 4 Department of Engineering, University of Technology and Applied Sciences, Nizwa 611, Oman; shabbirugce@gmail.com; 5 School of Mechanical Engineering, KLE Technological University, Hubballi 580031, India; patilarun7@gmail.com

**Keywords:** ABS, graphite, FDM, friction, wear, scanning electron microscopy

## Abstract

In this study, acrylonitrile butadiene styrene (ABS) and graphite powder—a solid lubricant—were filled and characterized for friction and wear responses. The fused deposition modeling (FDM) technique was utilized to synthesize ABS–graphite composites. A twin-screw extrusion approach was employed to create the composite filament of graphite–ABS that is suitable for the FDM process. Three graphite particle ratios ranging from 0% to 5% were explored in the ABS matrix. The wear and friction properties of ABS composites were examined using a pin on disc tribometer at varied sliding velocities and weights. As a result of the graphite addition in the ABS matrix, weight losses for FDM components as well as a decreased coefficient of friction were demonstrated. Furthermore, as the graphite weight percentage in the ABS matrix grows the value of friction and wear loss decreases. The wear mechanisms in graphite filled ABS composites and ABS were extensively examined using scanning electron microscopy and confocal microscopy.

## 1. Introduction

Polymers of many sorts have been used for both technical and industrial purposes. Recently, the requirements for polymer behavior and composite behavior for diverse attributes have been enhanced. Polymer property improvement can be realized by combining polymers and filler components to meet the application’s needs. Reinforced polymeric materials are the most appealing, and they are widely utilized in mechanical parts such as bearings, brakes, cams, bushes, gears, impellers, seals, and wheels [1,2,3].

In recent years, a wide range of reinforced polymers have emerged for use in advanced composite materials. Due to the high performance of light weight structures such as space vehicle frames and airframes, composite materials based on polymer have garnered a lot of attention for applications. Aside from their well-known capabilities in aircraft structures, reinforced plastic composites have come into existence to have stronger resistance to wear as well as low friction, which are required in particular specialized technical applications. As a type of key tribo-engineering material, polymer–matrix composites were revealing a huge amount of potentiality in industrial applications, not only because of their low unit cost and ease of manufacture, but also because of their capability for great tribological efficiencies in designed frames [4,5,6,7]. The presence of fillers helps to reduce the coefficient of friction while also improving wear resistance, as evidenced by several studies [5,6,7]. The solid lubricants or fibres that are contained in polymer composites are well recognized as tribo-materials and have been used on components that are meant to work without the use of external lubricants. Previously, the polymers’ wear resistance and mechanical strength were generally improved, but later on raises the characteristics of friction and aids in commanding the wear. The addition of nanoparticles to polymer composites can significantly improve their tribological properties.

Suresha and colleagues [8], using a pin-on-disc arrangement, compared the tribological properties of carbon-epoxy (C-E) and glass-epoxy (G-E) composites. The experiment is carried out by sliding the samples C-E opposite to a hard steel disc under various loading and sliding circumstances. This research presents the wear behavior of G-E and C-E composites as well as friction that operates at a set sliding distance. Whereas, regardless of the speed or load utilized, C-E composites display lower slide wear loss as well as lower friction than G-E composites. There are several additive manufacturing (AM) methods available, as well as a variety of price alternatives for pros and enthusiasts. FDM (fused deposition modeling), often known as material extrusion, is the most prevalent and widely used commercially available AM technology. FDM machines deposited stripes of melted thermoplastics such as PLA (polylactic acid) and ABS (acrylonitrile butadiene styrene) (ABS) to form a three-dimensional object. FDM parts have higher mechanical performance and are less expensive than other AM processes. FDM-ABS parts, for example, have the advantage of being both environmentally friendly and reusable. Aside from that, they have additional features, such as being light in weight and chemically inert. For FDM applications, ABS is thus well suited for the production of parts as well as tribological and mechanical applications [9,10]. The tribological characteristics of Nylon6-Al-Al_2_O_3_ and ABS components were compared by developing using the FDM process. At room temperature, under dry sliding settings, the FDM-built parts and their sliding wear behavior, as well as three different segments of Al_2_O_3_ and Al, were studied. The weights were applied at 5, 10, 15, and 20 N at a sliding velocity of 1.36 m/s and periods of about 5 and 10 min. The results show that FDM-built ABS counterparts have worse wear resistance, whereas FDM-built Nylon6-Al-Al_2_O_3_ components surpass them. Furthermore, the impacts of filler materials on distinct wear mechanisms, such as abrasion and adhesion, have been found. The composites created with different measurements were also demonstrated to be more wear-resistant, and the friction force and friction coefficients are lower in FDM components than in commercially available ABS [11]. Keshavamurthy et al. [12] produced ABS composites loaded with copper with the help of FDM (fused deposition modeling), and its wear behavior and friction have been evaluated. The filament of the copper–ABS composite had been produced utilizing the twin-screw extrusion process. ABS + 5wt% Cu, pure ABS, and ABS + 2.5wt% Cu are the three different materials that have been investigated. The wear properties and friction of copper filled ABS and pure ABS have been investigated at various sliding velocities and weights. The copper powder incorporation significantly improved the wear behavior and friction of the produced composites.

However, with the FDM approach, the parts that are manufactured have substantial limitations such as fragile functionality and a lack of strength for use as load bearing components as well as fully working. The limited wear resistance, brittle stiffness, and low heat conductivity of FDM parts further limit their utility as functional parts in a variety of technological applications [13].

On the other hand, graphite has remarkable lubricity, shock resistance, and corrosion resistance in a wide range of temperatures and environments, and at raised temperatures, it has higher strength retention and a high strength to weight ratio [14,15]. Many researchers have concentrated their efforts on improving the qualities of ABS-based components through the application of reinforcement. FDM is a promising alternative to traditional manufacturing methods, providing developers with extensive topological and geometrical freedom during the design process, as well as fast prototyping customization. The tribological properties of FDM surfaces, on the other hand, have yet to be fully studied.

The purpose of this research is to investigate the effect of graphite fillers on the tribological behavior of polymer composites. The effects of load, sliding speed, as well as graphite content on the friction and wear features of FDM components were thoroughly addressed.

## 2. Materials and Methodology

**Figure 1:**
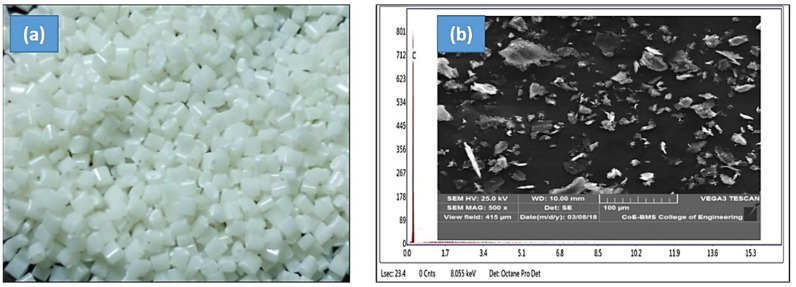
(**a**) Photograph of as received ABS pellets as initial material for twin screw extrusion and (**b**) scanning electron micrograph and EDAX spectrum of as received graphite powder showing the morphology and composition.

ABS (acrylonitrile butadiene styrene) was used as a matrix material. Figure 1a depicts a photo of ABS pellets that were used in the current investigation. For developing composite filament, ABS plastic was used as matrix, which was obtained in the form of pellets, as shown in the image. ABS plastic was chosen as a matrix material due to its wide range of applications in the industry sector.

Graphite powder was used as a filler material. In this study, 99.99 percent of pure graphite was used. The graphite particles ranged in size from 10 to 40 microns and were irregular in shape. M/s ACE Rasayan chemical agencies, Bangalore, INDIA, supplied the graphite powder. Figure 1b depicts the SEM graphite powder used in the experiment. Furthermore, graphite powder was subjected to EDAX analysis, which confirms the composition of the graphite.

### 2.1. Twin Screw Extrusion and Fused Deposition Modeling

Blending is accomplished by combining graphite filler material and ABS pellets. The plastic compounding machine was used to blend the graphite material as well as the ABS pellets in the prescribed composition, and the fitted necessary size was obtained for twin screw extruder. In order to achieve uniform dispersion of graphite particles in ABS pallets, the speed and process temperature were optimized with the use of various trails. On graphite-filled ABS pellets, twin screw extrusion was employed to make FDM filament with a diameter of 1.75 mm. To get homogenous measurements as well as an excellent surface quality suitable for FDM applications, the extrusion speed and temperature were optimized with varying trails. Filament for 3D printing was produced by combining various compositions containing graphite particles. All three compounds, namely 5% graphite filled ABS, 2.5 percent graphite filled ABS, and pure ABS, were produced for all three filaments. The FDM pieces were constructed to the proportions necessary for the friction and wear tests. Throughout the FDM process, all parameters were kept constant; the process parameters determined for building components are listed in Table 1. The FDM components were produced using a PRAMAN 3D printer.

### 2.2. Friction and Wear Test

The wear and friction qualities of FDM produced ABS and ABS-graphite parts were examined on a Pin-On-Disc Tribometer (Model: TR20LPHM-400). Manufacturer: DUCOM Instruments, Peenya, Bangalore, India). The experiments were carried out in accordance with the ASTM G99 test technique. The FDM process was used to produce samples with dimensions of 8 mm diameter and 20 mm length. Prior to each test, the test specimens’ surfaces were polished to a roughness of roughly 0.5 microns, cleaned with acetone-soaked cloth, and then dried. The counter surface disc is made of EN 24 steel and has a diameter of 160 mm, a surface roughness of 1 micron, and a thickness of 10 mm.

The pin assembly was first weighed using a digital electronic balance with a 0.1 mg precision. The test was carried out by adding a weight of 5 N–20 N and at varied sliding velocities of around 0.262, 0.524, 0.86, 1.05 m/s. At the end of the test, the pin assembly was weighed again in the same balance. The wear loss for each specimen was represented in terms of height loss data captured using linear variable differential transducer. Three repeated samples were evaluated for all the test conditions. At 1-min intervals, the friction force at the specimen’s sliding interface was measured using a frictional load cell. The friction coefficient was calculated by dividing the frictional force by the normal force applied using following Equation (1).

µ = F/N(1)

where, µ is coefficient of friction, F is frictional force (Newton) and N is applied load (Newton).

**Figure 2:**
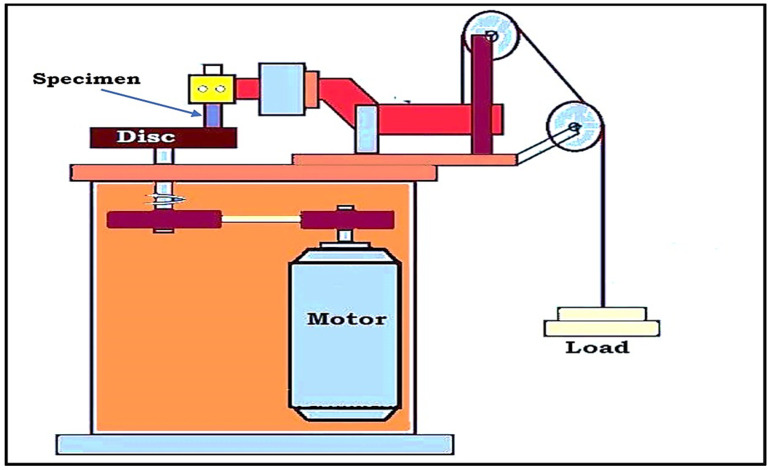
Schematic diagram of pin on disc machine (Tribometer) showing the details of specimen mounting and loading arrangements.

**Figure 3:**
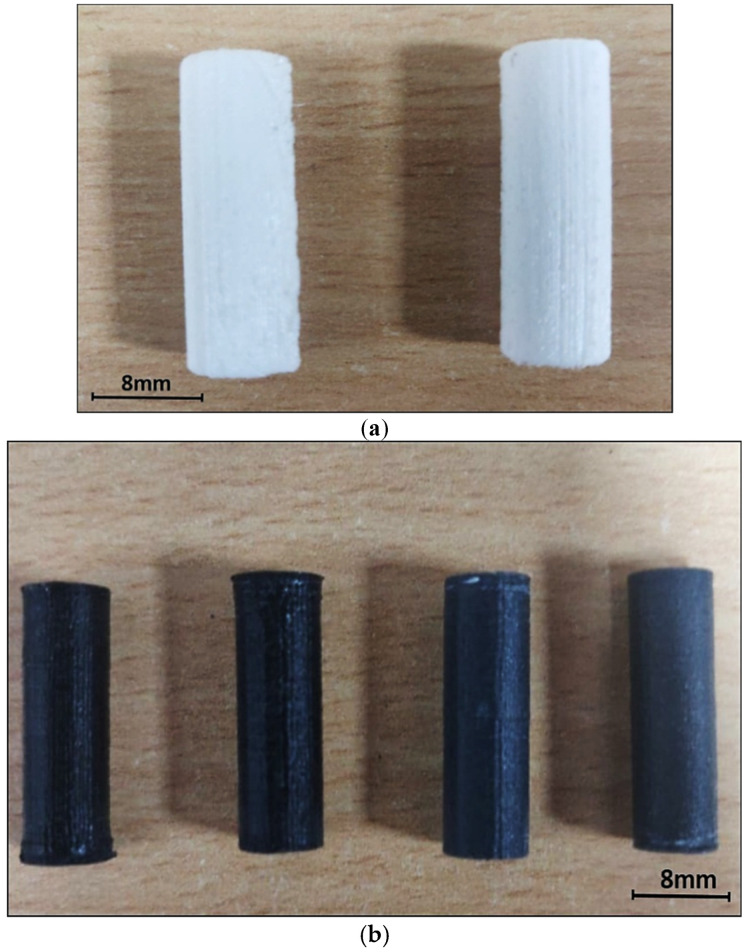
Photographs of specimens developed by FDM process for friction and wear test. (**a**) Unfilled ABS Specimens; (**b**) 5 wt% graphite filled ABS specimens.

Following the completion of the wear test, a scanning electron microscope was utilized to analyze the worn surface of the graphite filled and unfilled ABS parts. (Scanning Electron Microscope: TESCAN-VEGA3 LMU, Tungsten Heated Cathode, Probe Current: 1 pA to 2 μA). The gold coating was applied to the specimens that were evaluated in order to make them electrically conductive and so improve the resolution of the SEM observation. Figure 2 depicts the schematic diagram of friction and wear test machine, while Figure 3a,b depicts the photograph of the wear test specimens produced using fused deposition modeling. Confocal microscopy (CM) studies were performed using Olympus LEXT 4000 microscope to examine worn out surface morphology.

## 3. Results and Discussion

### 3.1. Influence of Graphite Content on Coefficient of Friction and Wear

**Figure 4:**
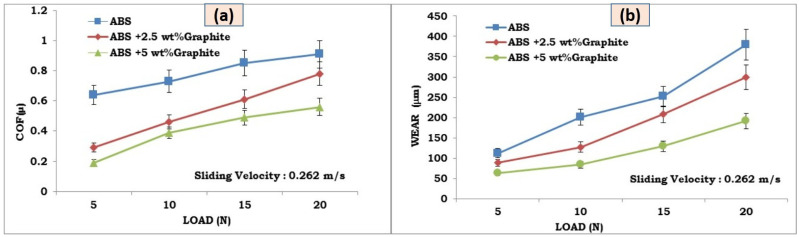
(**a**,**b**) Graphical representation of effect of load on coefficient of friction and wear loss for unfilled and graphite filled ABS parts developed by FDM process.

**Figure 5:**
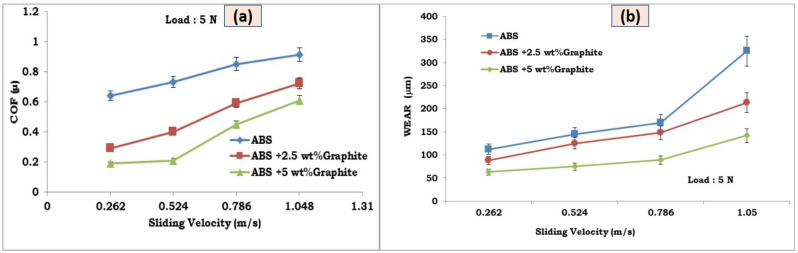
(**a**,**b**) Graphical representation of effect of sliding on coefficient of friction and wear loss for unfilled and graphite filled ABS parts developed by FDM process.

Sliding wear tests were used to study the effects of graphite on the wear and friction performance of ABS–graphite composites. Figure 4a,b and Figure 5a,b indicate the influence of graphite incorporation on the friction coefficient and wear behavior of 3d printed ABS parts, respectively. It is discovered that by incorporating graphite particles into the ABS matrix, the wear loss and friction coefficient are greatly reduced. For a given load value of 10 N and sliding velocity of 1 m/s, the average coefficient of friction of the ABS-5 wt% graphite and ABS-2.5 wt% graphite specimens is reduced by 44 and 31 percent, respectively. In the instance of wear loss, graphite-filled FDM specimens showed a 27% and a 55% reduction in wear loss under the same load and sliding velocity. It implies that the lubricant particle has a substantial impact on the wear behavior and friction of FDM composites. The use of graphite particles dramatically reduced the FDM composite friction coefficient. Most significantly, the friction coefficient of the graphite-filled ABS composites was lower (0.22) than that of the unfilled ABS sections (0.65) [16]. The figures show that adding graphite to FDM composites significantly enhances their tribological properties. Graphite owns a layer structure, with atoms in each layer grouped in a hexagonal unit cell. These layers are linked by weak van der Waals connections, which are easily destroyed by shear forces under sliding conditions [17].

The graphite’s beneficial effect on composite reduced Coefficient of friction(COF)as well as lower wear loss is due to graphite layers cleavage during loading, which is determined between layers of graphite by weak van der Waals bonds. More cleavage sites appear as the graphite concentration in the composite increases, resulting in a decrease in COF. Graphite is well-known for its structure, which consists of a stack of hexagonal plates on top of which the atoms give susceptible bonds to their surrounding areas. Moreover, it is claimed to be soft and brittle, making it a suitable solid lubricant; this indicates how wear loss and coefficient of friction are lowered. Similar behavior has already been seen in the case of various composites as well as polymers. The graphite’s crystalline lamellar structure allows the basal planes to glide against one another without dissolving, making it a strong solid lubricant. Lubricity is responsible for the existence of condensable vapors such as water, as well as van der Waals bonding forces between planes and C-C bonding between the carbon atoms of individual molecules. This excessive lubricity has been attributed to the incommensurability of the rotating graphite layers [18]. When comparing filled and unfilled ABS parts, the graphite particles provide greater wear resistance. The production of lubricating transfer layers of smeared graphite on the cast iron disc, as well as the first cleaning of each new track’s surfaces, are attributed to the rapidly decreasing friction coefficient and wear loss values. Further, addition of graphite particles contributes to improved bulk mechanical properties of the FDM parts. Increase in the ultimate tensile strength of the ABS–graphite composites developed by FDM process are reported in the previous works [19]. Enhanced mechanical properties of the FDM parts plays a vital role in reducing the wear loss of the test specimens. Addition of the graphite particles in the ABS materials acts as load bearing elements specifically at higher loads and sliding velocities and contributes to overall improvement in the wear characteristics of the FDM parts.

### 3.2. Influence of Load on Friction and Wear

Figure 4a,b depicts the fluctuation of COF and wear loss with respect to load for pure ABS and graphite reinforced ABS components, respectively. It was discovered that as load raises, so does COF. Components made of pure ABS and graphite reinforced ABS. Graphite reinforced FDM parts, on the other hand, had a reduced COF across the board. When compared to pure ABS, increasing the filler component reduces COF at all loads investigated. COF drops as load increases, which could be related to changes in wear mechanisms at higher loads. Excessive plastic deformation of FDM parts at higher loads causes a change in the wear mechanism and a drop in COF. However, the improved lubricating properties of the filler material play an important role in lowering COF even at greater loads. This clearly shows the tribological properties of graphite reinforced FDM pieces at greater stresses. During dry sliding, the heat created by the friction induced by material deformation in the true contacting point causes a rapid increase in adhesive friction. It was widely assumed that the component of friction resulting from adhesion was equal to the shear strength of polymeric composites as well as the product of the real contact area. As the load increased, so did the contact temperature, resulting in two opposing impacts on the coefficient of friction. The actual contact area increased at one end, followed by a rise in the friction coefficient. The shear strength, on the other hand, decreased, and as a result, the coefficient of friction decreased. As a result, the ultimate coefficient of friction would be determined by the two competing actions. It is possible to detect that the wear rate decreases as the load increases. The newly formed debris could form an even more unified but on worn surface a thinner layer beneath the high load, resulting in a decreased wear rate due to less abrasive wear of two bodies [20].

It is typically noticed that as the load increases, so does the wear loss for all three compositions. This could be attributed to more plastic deformation at higher loads. Increased plastic deformation causes surface cracking, resulting in the removal of a greater amount of material from the surface of the test sample. Furthermore, with greater loads, the interfacial film/transfer film present in the interface between the test sample and the steel disc becomes distracted and unstable, bringing the actual surface into contact, resulting in increased wear loss. However, in all of the scenarios analyzed, FDM components reinforced with graphite outperform unreinforced ones in terms of wear loss.

### 3.3. Influence of Sliding Velocity on Friction and Wear

Figure 5a,b depict the COF and wear loss variations of ABS filled graphite and ABS in terms of sliding velocity, respectively. The graph shows that the COF decreases as the sliding velocity increases for all of the compositions studied. Graphite filled FDM parts, on the other hand, show a considerable reduction in COF values when compared to pure ABS at all sliding speeds examined. As previously stated, an increase in COF with increased sliding velocity could be related to changes in the wear mechanism at higher sliding velocities. As the sliding velocity increases, the interfacial temperature between the test specimen and the steel disc rises, resulting in softening of the test specimen and excessive plastic deformation. At greater sliding velocities, this change in surface shape affects frictional values as well as wear behavior. Furthermore, in the case of graphite filled FDM parts, the greater thermal conductivity and good lubricating qualities of graphite particles limit interfacial temperature as well as the wear mechanism at the interface between test specimen and steel disc.

Figure 5 depicts the effect of sliding speed on the wear behavior of graphite-filled ABS composites. The graph clearly shows that the wear loss of graphite filled ABS composites is much lower than that of unfilled ABS parts. The wear loss of the FDM pieces increases as the sliding velocity increases for all of the compositions examined. However, as compared to unreinforced FDM components under similar test circumstances for all sliding velocities investigated, FDM parts reinforced with graphite powder exhibit much lower wear loss. As the sliding velocity increases, higher interface temperature may cause more wear loss. Temperature increases weakens the surface of test samples and increases wear loss. ABS composites loaded with graphite have better tribological characteristics than empty ABS composites at both high and low sliding speeds. As there was insufficient time to establish extra adhesive spots due to the lower contact time at greater sliding speeds, the adhesive friction force was reduced. Furthermore, the transfer film is simple to manufacture and difficult to rupture, resulting in enhanced tribological properties at higher sliding speeds. The debris of solid lubricant can form a ‘third body interface’ very quickly after crushing them in the contact zone, under high sliding speed and high load, and as a result, the wear mechanism changed from solid body friction to dry lubricated friction [21,22].

### 3.4. Worn-Out Surface Analysis

**Figure 6:**
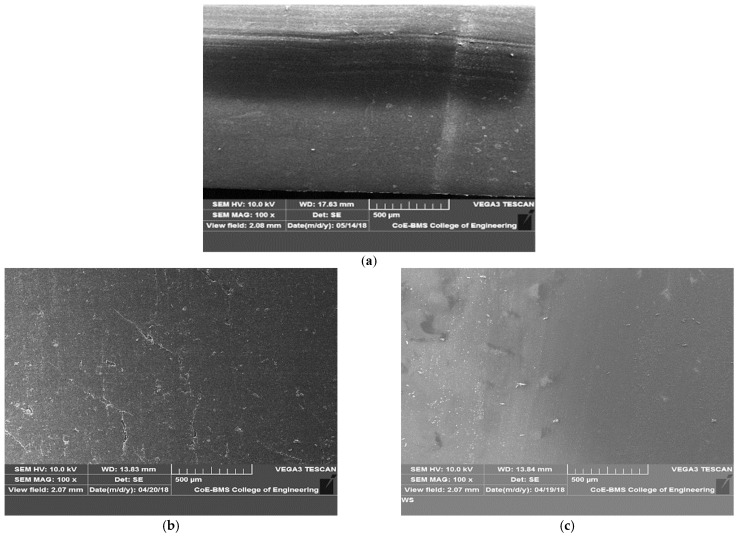
Scanning electron micrographs of the wear test specimens showing the morphology the surfaces before the friction and wear test. (**a**) Pure ABS; (**b**) ABS + 2.5 wt% graphite; (**c**) ABS + 5 wt% graphite.

**Figure 7:**
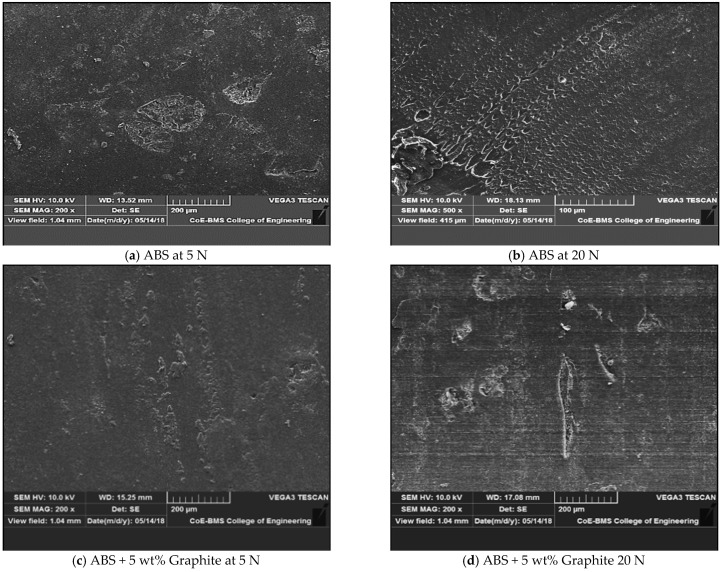
Scanning electron micrographs of the wear test specimens showing the morphology the worn out surfaces after the friction and wear test at different loads. (**a**) ABS at 5 N; (**b**) ABS at 20 N; (**c**) ABS + 5 wt% graphite at 5 N; (**d**) ABS + 5 wt% graphite 20 N.

Figure 6 depicts the surfaces of graphite-filled ABS plastics and pure ABS prior to the friction and wear test. Figure 7 depicts the worn-out surfaces of graphite-filled ABS plastics and pure ABS evaluated at various loads. SEM of the specimens were captured before the wear test for the purpose of the comparison. It was discovered that both pure ABS and graphite infused ABS display surface deterioration as the sliding velocity and load are raised; with an increase in the load, the damage level increases. However, with all sliding speeds and loads examined, the amount of surface damage found in graphite filled FDM parts is significantly less than that observed in pure ABS. In the case of pure ABS, there is severe plastic deformation. There are no visible surface damages or cracks in graphite filled FDM parts, which may be attributed to improved physical and mechanical properties of FDM parts due to the presence of graphite, as well as improved surface hardness and lubrication characteristics, which are significantly attributed to the reduction of wear loss. The surface shape of over note surfaces clearly reflects the experimentally observed tribological tendency.

**Figure 8:**
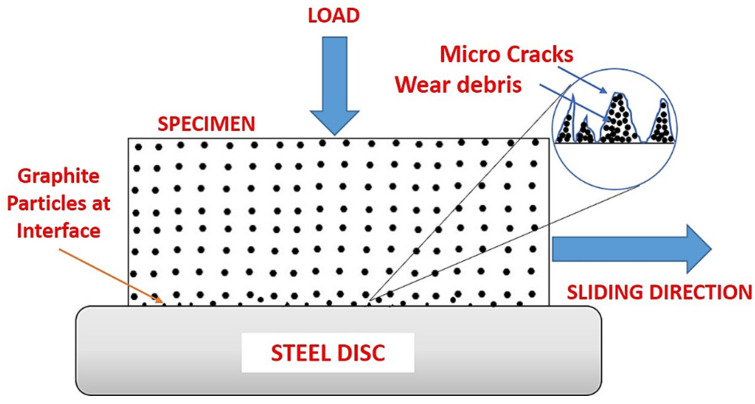
Schematic representation of the wear mechanism involved in the graphite filled ABS samples developed by FDM process.

Many exposes and defects were seen on the worn surfaces, resulting in significant wear for unfilled composite. The wear of graphite filled composites is less severe than that of unfilled composites. Due to the increased load-carrying capacity offered by graphite particles, the worn surface has a layered structure with fissures. The wear damage on the worn surface of graphite filled composites was found to be the mildest, with almost no matrix separation and few cracks. The presence of graphite particles is thought to be responsible for the filled composite’s improved tribological characteristics. Figure 8 depicts the wear model of a graphite-filled composite. At one end, a favorable rolling action induced by microscopic particles reduces the coefficient of friction; at the same time, tiny wear debris is generated, which can fill in gaps and holes, resulting in a smooth worn surface. On the other hand, the improved load carrying capacity supplied by graphite particles significantly reduced extreme abrasive wear during the sliding process, resulting in better mechanical characteristics of the composite components when paired with groove-filling debris.

**Figure 9:**
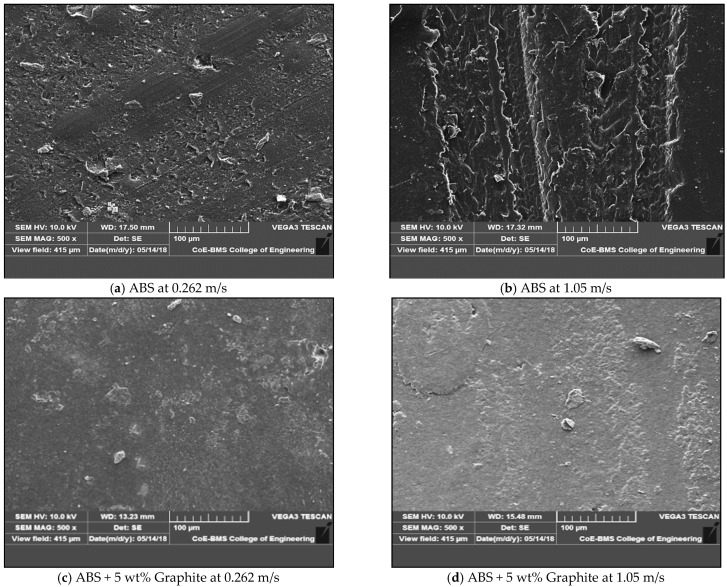
Scanning electron micrographs of the wear test specimens showing the morphology the worn out surfaces after the friction and wear test at different sliding velocities; (**a**) ABS at 0.262 m/s; (**b**) ABS at 1.05 m/s; (**c**) ABS + 5 wt% graphite at 0.262 m/s; (**d**) ABS + 5 wt% graphite at 1.05 m/s.

Figure 9 illustrates SEM morphologies of worn surfaces as well as graphite filled FDM components and unfilled at different sliding velocities. The worn surfaces of the unfilled FDM pieces were rough, with a lot of scuffing and plastic deformation. Some of the wear debris was discovered on the counterpart, and while the transfer film was thick, it was not constant in thickness, implying that excessive adhesive wear had gained precedence. The scuffing impact was reduced because the worn surfaces of the graphite-filled FDM parts were rather smooth, and the transfer layer was thick and continuous, but not uniform, indicating that fatigue wear as well as adhesive took the dominant position. Sliding occurred as a result of the development of a coherent transfer film and more uniformity, and between the surfaces of the transfer film as well as the graphite filled composite, thereby minimizing the contact directly. As a result, a decrease in coefficient of friction and wear rate is unavoidable. Under high velocity, plastic deformation was detected on the worn surface of graphite-filled FDM parts. There was a lot of small wear debris on the worn surface, and the transfer layer was thick but not evenly dispersed. At 0.262 m/s sliding, it was possible to see a well-integrated layer on the worn surface. As the load increased, some of the large particles shaped, or in the wear surface the flaky debris sheared or shattered into thinner flakes or tiny particles as well as functioned as lubricants; on the worn surface, the freshly produced debris could generate a much more unified layer and also the direct contact between the counterpart and the filled FDM component, therefore, even at high sliding velocity as well as at high load, a decrease in the wear rate as well as coefficient of friction were unavoidable.

Both at different loads and sliding speeds, the SEM pictures show the ABS specimen after it was subjected to a 10-min wear test at a load of 20 N. Ploughing, long continuous grooves, micro-cutting, and so on are all evidenced by the wear surface, and all of this is due to abrasion under both loads and sliding speeds. De-lamination occurs when some layers of material are removed from the surface; as the velocity and load increase, so does the severity of the de-lamination. The heat generated at the sliding surface interface tends to cause plastic deformation of the worn material. Plastic deformation increases with increased load. The major wear mechanism is thus surface delamination and abrasion, with only a few adhesions and plastic deformation involved. Some abrasive wear characteristics, including ploughing, micro-cutting, and grooving, can be visible on the worn surface. The appearance of the surface is rather bright and clean, with little evidence of wear. Aside from abrasive wear, plastic deformation and surface de-lamination appear to be greatly reduced in these specimens. This is due to the composite material’s thermal stability as well as its better load bearing capacity when compared to ABS material. Furthermore, because of the aforementioned mechanisms, the wear impact is decreased in the composite matrix by graphite augmentation.

### 3.5. Confocal Microscopy

**Figure 10:**
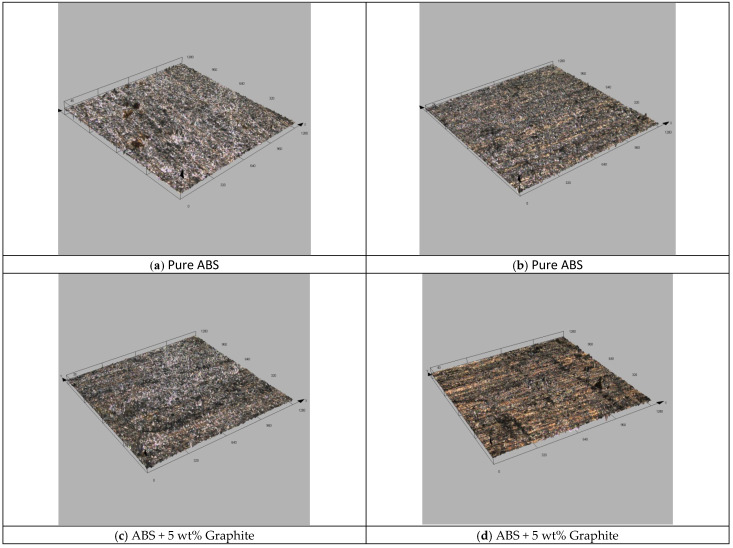
Three dimensional confocal images of worn-out surfaces showing the morphology of the worn-out surfaces.

Figure 10 depicts confocal microscopic images of the composites’ deteriorated surface. The graphite-filled composite had the thinnest wear scar and the smoothest worn surface, which corresponded to the lowest wear rate. By increasing the graphite content to 5% wt, the composite’s wear rate and coefficient of friction both decreased significantly, indicating that the wear as well as characteristics had improved. The smallest wear rate corresponds to the thinnest wear scar and the smoothest worn surface in 3D microscopic pictures. It is claimed that as graphite content increases, the composite’s load-carrying performance improves due to the increased impact of interlayer slip in multilayer structured graphite, resulting in less severe wear damage.

## 4. Conclusions

This study examined the tribological properties of graphite-filled ABS-based FDM parts in a systematic manner. The following conclusions can be derived from the findings of this study.

Increasing the graphite content in FDM components reduces wear loss and friction coefficient significantly at all sliding velocities and loads. The coefficient of friction is lowered from 0.65 to 0.22 as a result of the 5 wt% graphite powder composition. The wear rate dropped from 115 to 62 at 0.262 m/s sliding velocity and 5 N load, respectively.

Due to the presence of graphite, composite materials have a higher wear resistance than ABS materials. Furthermore, it was discovered that for composite materials, the wear processes of more than one contribute to wear loss. Surface delamination and abrasion are the most common mechanisms for ABS material wear; however, in the case of composite materials, wear is also produced by adhesion. Under all working circumstances, the graphite reinforced ABS material has a lower frictional coefficient and less material loss than unfilled ABS.

As the sliding velocity and applied force increase, the friction coefficient decreases for all of the compositions examined. However, under identical test settings, graphite-filled ABS parts show a considerable drop in COF due to the presence of graphite particles, which act as a lubricant between the test specimens and the steel disc.

## Figures and Tables

**Table 1:** Process parameters.

Orientation	X, Y, Z Directions
Layer height	0.1 mm
Fill density	100%
Speed	5 mm/s
Extrusion temperature	245 °C
Nozzle	0.3 mm
Bed temperature	90 °C

## Data Availability

Not applicable.
